# TERTmonitor—qPCR Detection of *TERTp* Mutations in Glioma

**DOI:** 10.3390/genes14091693

**Published:** 2023-08-25

**Authors:** João Paulo Brás, Tito Teles Jesus, Hugo Prazeres, Jorge Lima, Paula Soares, João Vinagre

**Affiliations:** 1 U-Monitor Lda, 4200-135 Porto, Portugal; joao.bras@uromonitor.com (J.P.B.); hprazeres@ipatimup.pt (H.P.); psoares@ipatimup.pt (P.S.); 2 Instituto de Investigação e Inovação em Saúde (i3S), Universidade do Porto, 4200-135 Porto, Portugal; tjesus@ipatimup.pt (T.T.J.); jlima@ipatimup.pt (J.L.); 3 Instituto de Patologia e Imunologia Molecular, Universidade do Porto (Ipatimup), 4200-135 Porto, Portugal; 4 Faculdade de Medicina, Universidade do Porto (FMUP), 4200-319 Porto, Portugal

**Keywords:** telomerase promoter, qPCR, genotyping, gliomas, CNS tumours, TERTmonitor

## Abstract

Telomerase promoter (TERTp) mutations are frequently observed in various types of tumours and commonly characterised by two specific hotspots located at positions −124 and −146 upstream of the start codon. They enhance *TERTp* activity, resulting in increased TERT expression. In central nervous system (CNS) tumours, they are integrated as biomarkers, aiding in the diagnosis and with a role in prognosis, where, in some settings, they are associated with aggressive behaviour. In this study, we evaluated the performance of TERTmonitor for *TERTp* genotyping in a series of 185 gliomas in comparison to the traditional method, Sanger sequencing. Against the gold-standard Sanger method, TERTmonitor performed with a 97.8% accuracy. Inaccuracy was mainly due to the over-detection of variants in negative cases (by Sanger) and the presence of variants that can modify the chemistry of the probe detection. The distribution of the mutations was comparable to other series, with the −124 being the most represented (38.92% for Sanger and TERTmonitor) and more prevalent in the higher-grade tumours, gliosarcoma (50.00%) and glioblastoma (52.6%). The non-matched cases are debatable, as we may be dealing with the reduced sensitivity of Sanger in detecting rare alleles, which strengthens the use of the TERTmonitor. With this study, we present a reliable and rapid potential tool for *TERTp* genotyping in gliomas.

## 1. Introduction

Telomerase promoter *(TERTp*) mutations are common in numerous types of cancers and, in particular, in those originating from slow-replicating tissues. *TERTp* mutations were firstly reported in melanoma [1,2] and, afterwards, described in several other cancers, such as hepatocellular carcinoma [3], thyroid carcinoma [4,5], bladder carcinomas [5,6,7], and central nervous system (CNS) tumours [5,6,8]; for a comprehensive review, please refer to [9]. Contrasting with the low reported frequency of mutations occurring in the coding region of the *TERT* gene, there are frequent mutations in the *TERTp* [10]. These mutations are most commonly located at two specific hotspots, at positions 1,295,228 and 1,295,250 on chromosome 5, positioned at −124 and −146 base pairs upstream from the *TERTp* ATG start codon, although other rare locations in the promoter are described [1,2]. These alterations lead to enhanced *TERTp* activity and are associated with increased TERT expression [5,11]. The presence of *TERTp* mutations in CNS tumours is highly frequent [10,12]. In fact, *TERTp* mutations have been integrated as key biomarkers in diagnostic procedures for primary CNS tumours, as recommended by the World Health Organization (WHO) guidelines, and it has an impact on the prognosis of glioma patients [13,14,15]. *TERTp* mutations are frequent in WHO grade II or III astrocytic gliomas and grade IV isocitrate dehydrogenase (IDH)-wildtype glioblastoma with an aggressive behaviour. They are frequently associated with *IDH*-wildtype gliomas in combination with gain of chromosome 7 and loss of chromosome 10 (+7/−10) or *EGFR* amplification; exclusion of +7/−10 or EGFR amplification events do not preclude aggressive clinical behaviour in some cases with *TERTp* mutations [16,17,18,19]. For *IDH*-wildtype glial neoplasms without WHO grade IV histology or aggressive behaviour, we and others have also demonstrated occasional *TERTp* mutations in tumours classified as pleomorphic xanthoastrocytoma, ganglioglioma, anaplastic glioma with pilocytic features, and ependymoma [5,20,21,22]. *TERTp* mutations are frequent in oligodendrogliomas, with peak frequencies of 74–78% [20], and are often associated with other molecular alterations [8,20]. Understanding the frequency and association of *TERTp* mutations is important for molecular classification and for potential targeted therapies, as unveiled for the prognostic interaction of *TERTp* mutations with *MGMT* promoter methylation in *IDH*-wildtype glioblastoma patients, who presented a better outcome when treated with temozolomide [23,24]. In this study, we repurposed a commercially available kit, Uromonitor^®^, which is designed for the genotyping of *TERTp*, *KRAS,* and *FGFR3* hotspot mutations, using qPCR technology, in the detection of non-muscle invasive bladder cancer recurrence using urine. Uromonitor^®^ has been extensively evaluated in multicentric studies [25,26] and in a clinical trial (NCT03962933: urine-based detection of non-muscle invasive bladder cancer (SOLUSION)),with recent impactful results for patients and clinicians [27]. For this purpose, we used the proprietary component of Uromonitor^®^ that targets *TERTp* genotyping, hereby named TERTmonitor, and evaluated its performance in gliomas against the standard analysis method, Sanger DNA sequencing. For a better characterization, we evaluated a series of 185 gliomas with different histotype compositions. With this approach, we aimed to evaluate if TERTmonitor can be considered a potential fast and reliable tool for *TERTp* genotyping in gliomas.

## 2. Materials and Methods

### 2.1. Tumour Samples

Representative formalin-fixed paraffin-embedded (FFPE) tissue samples from 185 gliomas were retrieved from the pathology archives of a Portuguese institution, Centro Hospitalar e Universitário de São João (CHUSJ). All tumours were previously reviewed and classified according to the WHO classification of CNS tumours [28]. This cohort included cases classified as pilocytic astrocytoma (*n* = 8), diffuse astrocytoma (*n* = 29), oligodendroglioma (*n* = 29), anaplastic oligodendroglioma (*n* = 37), glioblastoma (*n* = 76), and gliosarcoma (*n* = 6). All procedures described in this study were in accordance with national and institutional ethical standards and previously approved by CHUSJ Local Ethical Review Committee: project nº 64/2014 *“Estudo de mutações nos genes IDH1 e IDH2 e no promotor do gene TERT em gliomas humanos: correlação com vias metabólicas e características clinicopatológicas”*. Patient informed consent was not required due to being an anonymized retrospective study as in accordance with Portuguese legislation.

### 2.2. Genotyping Characterisation

#### 2.2.1. DNA Extraction

DNA was obtained from 10 µm dissected sections of FFPE tissues. Sections were deparaffinized in xylene, followed by incubation in alcohol. Tissue was carefully scraped to a microcentrifuge tube. DNA was isolated using column capture process using the Ultraprep Tissue DNA Kit (AHN Biotechnologie, Köln, Germany) following manufacturer’s instructions. The extracted DNA was quantified via spectrophotometry using a Nanodrop ND-1000 (ThermoScientific, Waltham, MA, USA), and quality was assessed through analysis of 260/280 and 260/230 nm ratios.

#### 2.2.2. Amplification and Sanger Sequencing

Amplification of genomic DNA (25–100 ng) was performed with PCR using Qiagen Multiplex PCR kit (Qiagen, Hilden, Germany). For PCR genotyping of the −124 G>A and the −146 G>A hotspot *TERTp* mutations, the following primer pairs were used: Fw: 5′-CAGCGCTGCCTGAAACTC-3 and Rv: 5′-GTCCTGCCCCTTCACCTT-′3 (according to the manufacturer’s instructions). The obtained 235 base pair product was then direct sequenced using Sanger method with the ABI Prism BigDye Terminator Kit (Life Technologies, Carlsbad, CA, USA). The fragments were run in an ABI prism 3500 xL Genetic Analyser (Life Technologies). The sequencing reaction was performed in forward direction. An independent confirmatory PCR amplification/sequencing, both in a forward and reverse direction, was performed in positive samples or samples that were inconclusive.

#### 2.2.3. TERTmonitor

For TERTmonitor, detection of the −124 G>A and −146 G>A mutations in *TERTp* was conducted on 25 ng of the extracted DNA; the same DNA samples used in Sanger genotyping were evaluated using qPCR. Analysis was carried out on a StepOne Plus qPCR instrument (Applied Biosystems, Waltham, MA, USA) using the proprietary chemistry for amplification and detection of *TERTp* mutations, as partially provided with the Uromonitor^®^ (U-Monitor Lda, Porto, Portugal). Briefly, two independent multiplex reactions were prepared: (a) *TERTp* −124 G>A mutation (mut) with FAM (blue plots) and *TERTp* −124 wildtype (wt) with HEX (green plots), and (b) *TERTp* −146 G>A mut with FAM and *TERTp* −146 wt with HEX. Mixes were prepared as described by the manufacturer by adding the components provided, including the master mix and the respective set of primers and probes for detection of the hotspot mutations, as well as the respective wt controls for quality assessment. TERTmonitor primers and probes use a patented technology (sequences are not provided) with high sensitivity and specificity for the target sequences. Plates were set up by adding 9 μL of mix and 1 μL of DNA (25 ng). Multiplex qPCR runs were performed as follow: 1 cycle at 95 °C for 3 min followed by 50 cycles at 95 °C for 20 s and 68 °C (TERT −124)/64 °C (TERT −146) for 45 s. Amplification signals in FAM and HEX channels were acquired and analysed as recommended by the manufacturer, using Design and Analysis software 2.5 (Applied Biosystems) based on multicomponent plot representations. If at least one of the screened alterations provided a positive result in replicates, then the test was positive. For inconclusive signals in replicates, a novel independent reaction was generated; if two positive signals were obtained in different reactions, a positive result was considered. Positive and negative control samples were included for assay’s validity.

### 2.3. Statistical Analysis

Representative Statistical analysis was conducted with IBM^®^ SPSS^®^ Statistics for Mac, version 24.0 (IBM, Chicago, IL, USA). The results are expressed as a percentage. Figures were done in GraphPad Prism v9.0. for Mac.

## 3. Results

The presence of *TERTp* mutations was detected in 101 out of the 185 (54.6%) gliomas via Sanger sequencing. Using the TERTmonitor, 103 mutations were detected in the 185 gliomas (55.7%). For both analyses, the same DNA samples were used. Stratification of the mutations and overall frequency by histotype are presented in Table 1.

In this study, lower frequencies of *TERTp* mutations were detected in pilocytic astrocytoma (WHO grade I, with no detectable mutations (0.0%)) and in diffuse astrocytoma (WHO grade II), with an average frequency of 15.5% (13.8% via Sanger sequencing and 17.2% with TERTmonitor). For oligodendrogliomas (WHO grade II) and anaplastic oligodendrogliomas (WHO grade III), *TERTp* mutation frequencies obtained were of 55.2% and 65.2%, respectively. Higher values were detected in glioblastoma (WHO grade IV), with an average frequency of 69.1% (68.4% and 69.7% through Sanger and TERTmonitor, respectively). The highest frequency of *TERTp* mutations was detected in gliosarcoma (WHO grade IV), where all cases were positive and supported by both methods.

**Figure 1:**
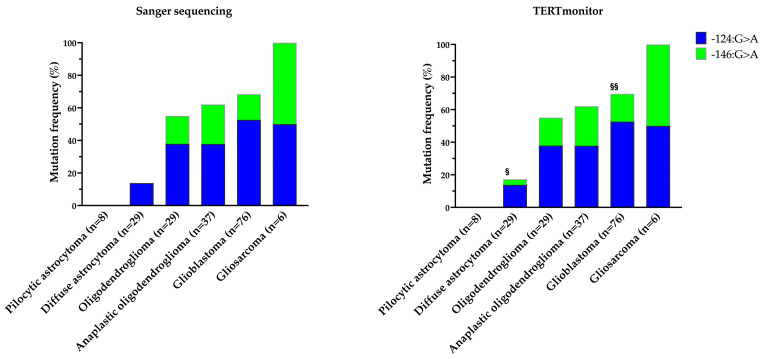
Comparison of Sanger sequencing and TERTmonitor—frequencies and type of *TERTp* mutations in the different histological subtypes of the gliomas. § detection of two additional mutated cases (−124 G>A and −146 G>A, respectively) and failure to detect one mutation (−124 G>A) in a case with TERTmonitor in diffuse astrocytoma; §§ detection of an additional mutated case (−146 G>A) of glioblastoma.

Taking Sanger sequencing as the gold standard and including positive and wildtype cases, the overall study accuracy was 97.8%. Analysing by histological subtypes, pilocytic astrocytoma, oligodendroglioma (including anaplastic), and gliosarcoma presented full concordance between the two methods. For the diffuse astrocytoma and glioblastoma, the accuracy was 89.7% and 98.7%, respectively. Overall, four unmatched cases were obtained and corresponded to samples of diffuse astrocytoma (*n* = 3) and glioblastoma (*n* = 1), as represented in Figure 1. In Figure 1, a stepwise increase in *TERTp* mutations with the increased grade of the tumours was also visible, in accordance with already extensive data published on glioma series in the literature [12,29,30].

In diffuse astrocytoma, TERTmonitor failed to report a −124 G>A mutated case previously identified with the Sanger sequencing method. In contrast, TERTmonitor identified two novel alterations, one in the −124 G>A and another in the −146 G>A hotspots, in two different diffuse astrocytoma cases, previously missed by Sanger sequencing and classified as *TERTp* wildtype. An additional −146 G>A mutated glioblastoma case, not identified via the Sanger sequencing method, was detected by the TERTmonitor.

**Figure 2:**
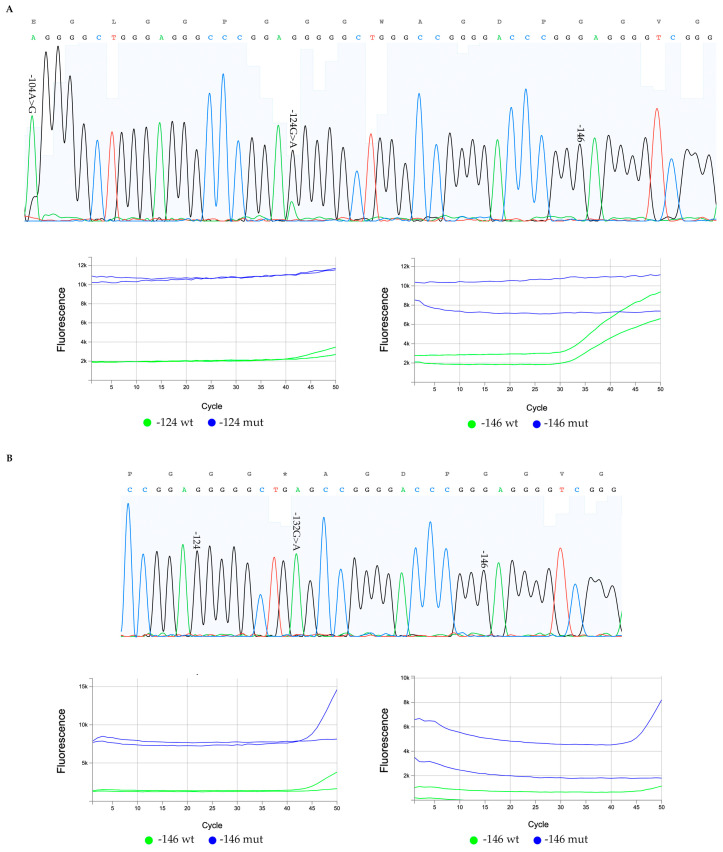
Sequencing chromatograms and qPCR multicomponent plots of glioma mutated cases with non-concordant results by Sanger method and TERTmonitor. (**A**) A non-concordant diffuse astrocytoma case with a hotspot −124 G>A heterozygous mutation detected in the sequencing chromatogram, as well as a rare variant, the −104 A>G (**A**, top). The corresponding TERTmonitor qPCR multicomponent plot (**A**, bottom left) for the −124 position fails to detect the −124 G>A mutation ((**A**) bottom left plots (blue, FAM)). An assay poorer performance is also perceivable in the −124 wt plots (bottom left, green (HEX)) where the late amplification of the wt sequences may be indicative of probe interference; and (**B**) a glioblastoma case with a −146 G>A mutation detected by TERTmonitor that is not present in the Sanger sequencing chromatograms. An additional alteration is present in the sequencing chromatogram, a −132 G>A rare variant in homozygosity (**B**, top chromatogram) that generates a novel motif in the opposite strand in reverse complement (CCGGCT) and partially complementary to the well-described *TERTp* mutations motifs. The bottom component plots (**B**) correspond to two independent runs for the −146-hotspot evaluation; for each run, in one of the replicates there is false detection of the −146 mutation (mut, blue, FAM), and late (or failed) amplifications in both plots, and in the different runs, indicative of probe interference.

Following the detection of unmatched results of mutated glioma cases between the application of the Sanger sequencing method and the use of TERTmonitor, we next sought to determine if we could decipher the reasons for these non-concordant cases. For this purpose, we pulled out the chromatograms of the sequences and the results from the correspondent qPCR runs, in particular, the multicomponent plots, as they are more informative than the amplification curves and are recommended by the manufacturer. We start by presenting cases where TERTmonitor differs from the Sanger sequencing method and present additional genomic alterations that may tamper probe function, Figure 2.

In one of the diffuse astrocytoma non-concordant cases, as presented in Figure 2, the *TERTp* chromatogram (Figure 2A) obtained using the Sanger sequencing method holds a −124 G>A hotspot mutation and a rare variant at the position −104 with a nucleotide change A>G; this rare variant creates a novel genomic motif (AGGGGCTGGG). This novel motif may tamper qPCR TERTmonitor probe performance, as it creates an identical motif present in the juxtaposed −124 G>A region. A possible interference is also perceivable in the multicomponent plot for the −124 wt assay (Figure 2A, bottom left, green curves (HEX) for each replica), where the performance of the probe in the run is reduced, taking into consideration the late amplification. 

In Figure 2B, in a glioblastoma case, we also detected a new −132 G>A alteration (Figure 2B, top) using the Sanger method in an apparent homozygosity that leads to the formation of a new motif AGCCGG. This motif generated in the opposite strand in reverse complement (CCGGCT) is, in part, complementary to the well-described *TERTp* mutation CCGGAA motifs; this motif is also responsible for the ETS family of transcription factors [1,2,5,31,32].

Additionally, the novel motif is, in part, complementary to the −146 probe; the full-sequence probe is not disclosed due to proprietary rights. In this case, the qPCR multicomponent plots (Figure 2B, bottom) for each run, we observed that one of the replicates falsely detects the −146 mutation (mut, blue, FAM), and late (or failed) amplifications in both plots, and in the different runs, once again, as in the first case, are indicative of probe interference. Overall, we considered that two of the four cases with a discordant result may be due to interference with the genotyping probes, giving, therefore, false results and representing rare events (1.1%—2 out of the 185 total cases). 

**Figure 3:**
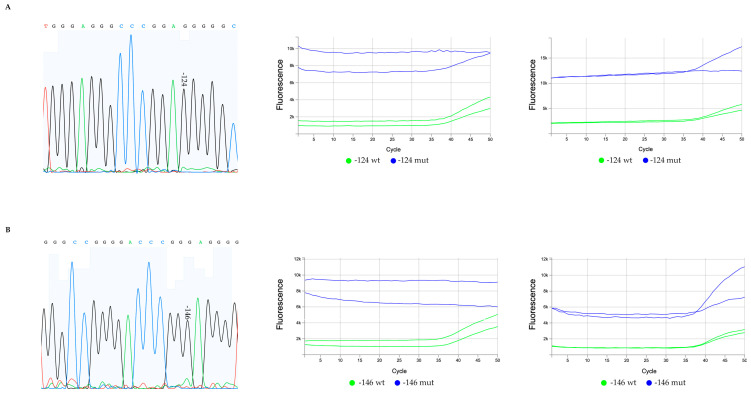
Sequencing chromatograms and qPCR multicomponent plots of mutated diffuse astrocytoma cases only detected by TERTmonitor. (**A**) A diffuse astrocytoma with a −124 G>A mutation not detected by Sanger sequencing (**A**, left) and the corresponding two independent qPCR TERTmonitor runs (with 2 replicates) (**A**, middle and right); whereas in both analysis the assays for the −124 mutation were not concordant for the mutation detection; (**B**) a −146 G>A diffuse astrocytoma mutated case detected by TERTmonitor that after re-evaluation of sequencing chromatogram, a small peak at the −146 hotspot was considered (initially considered an artifact) (**B**, left); in one of the qPCR TERTmonitor runs, the presence of the mutation was more clear (**B**, right). The initial qPCR run failed to detect the mutation (**B**, middle).

For the two remaining cases of diffuse astrocytoma with non-concordant results between the two methods, we encountered a different setting, where TERTmonitor detected the presence of variants not clearly evident through the Sanger sequencing method, Figure 3. 

In the first, Figure 3A, the −124 G>A mutation was not detected using Sanger sequencing by analysing the chromatogram (Figure 3A, left), but the two corresponding independent qPCR TERTmonitor runs (with two replicates in two independent runs) (Figure 3A, middle and right) revealed a marginal presence of −124 G>A mutation in one out of two replicas for each run. In the second case, Figure 3B, classified by the Sanger sequencing method as negative for −146 G>A mutation (Figure 3B, left), two corresponding independent qPCR TERTmonitor runs (with two replicates) were performed, and the presence of −146 G>A mutation was detected for both replicas in one out of the two independent runs (Figure 3B, right). For these two cases, and based on the sequencing chromatogram results, we considered that we may be detecting allelic fractions close to the limits of detection of the assay.

## 4. Discussion

*TERTp* mutations are frequently detected across several types of cancers and particularly highly represented in tumours derived from cells with low rates of self-renewal [1,2,5,8]. The two most common *TERTp* hotspot mutations are located at positions −124 and −146 upstream from the *TERTp* translational start site; the nomenclature of these mutations can also be present in reference to the original genome coordinates (1,295,228 and 1,295,250), being equivalently named 228 (−124) and 250 (−146). *TERTp* mutations are currently incorporated as significant biomarkers in the diagnostic protocols for CNS tumours, as advised by the guidelines of the WHO, and have a notable influence on the prognosis of patients with glioma [13,14,15].

In this study, we evaluated TERTmonitor performance in comparison to the gold-standard method, Sanger sequencing, in the evaluation of *TERTp* mutation genotyping. For this comparison, we used a series of gliomas previously characterised by Sanger. Gliomas are extensively characterized for *TERTp* mutations. They are known to present increasing accumulation of *TERTp* mutations with higher grades and, most importantly, are stratified for diagnosis and prognosis upon their mutational status [8,16,17,33,34,35]. In this analysis, we used a glioma series that is in line with most of the published data in terms of *TERTp* mutation frequency and number of cases distribution per histotype, in order to evaluate TERTmonitor in a real setting (for comprehensive reviews [10,12]). The series presented the highest frequency of *TERTp* mutations in gliosarcoma, where all cases were mutated and corroborated by both methods. Glioblastoma had a frequency of 69.1%, in accordance with the values around 70% described in the literature [12]. The average mutational frequency when considering both methods for oligodendrogliomas and anaplastic oligodendrogliomas was 62.2% and 55.2%, respectively, and corresponded to previously reported lower frequencies of 74.0% and 77.0% [12], respectively. Similarly, the lowest frequency of *TERTp* mutations was found in diffuse astrocytoma, with an average frequency of 15.5% (19–25% described [12]) and absent in pilocytic astrocytoma.

The mutation detection through qPCR multicomponent plot analysis was based on the major hotspot mutations (−124 and −146 G>A) screening, using a mutant allele-specific primer with TaqMan^®^-based technology probes for the real-time detection of nascent amplicons. The main goal was to evaluate a repurposed part of a genotyping kit, Uromonitor@, as a fast and effective method for improving detection thresholds, enhancing the ability to detect a minimal mutated allele fraction. In fact, TERTmonitor was revealed to have a high accuracy since, taking the Sanger sequencing as the gold standard, the study accuracy was 97.8%. In a previous report, Uromonitor^®^ test accuracy in FFPE tissue samples of non-muscle-invasive bladder cancer and, in comparison, to the Sanger sequencing method, achieved 98.5% for the *TERTp* −124 assay and 99.5% for the *TERTp* −146 assay [26]. From this study, the differences in accuracy between TERTmonitor and Sanger sequencing were mainly due to two pitfalls: (i) possible probe chemistry interference and (ii) reaching the limits of detection. The setting where a possible chemistry interference that could reduce the probe’s performance was revealed in two cases from this series. First, a diffuse astrocytoma −124 G>A mutated case that presented a rare variant detected via Sanger sequencing, −104 A>G. This variant creates a genomic motif similar to the juxtaposed −124 G>A region that may tamper TERTmonitor probe’s function and what was detectable in the qPCR run multicomponent plots. The second, a glioblastoma case with a −132 G>A alteration in the sequencing chromatogram reveals a new motif, which is, in part, complementary to the −146 probe. Probe interference is hard to control or predict, but we need to take into consideration that these false results represented rare events (1.1%) in the series. Still, a trained or experienced technician evaluating multicomponent plots would clearly identify a poorer assay performance and, in this case, Sanger sequencing would be recommended and would have resolved these situations. So, quality control is a mandatory step in the process and can easily circumvent this technicality.

The second setting, where we may be dealing with reaching the limits of detection, was revealed in two cases of diffuse astrocytoma, with non-concordant results between the Sanger sequencing method and TERTmonitor. One case with a −124 G>A mutation was not detected by Sanger but was detected by qPCR TERTmonitor runs. And, in another case with a 146 G>A mutation that was initially considered an artifact via the Sanger method, it was detected by the TERTmonitor. Evaluating the sequencing chromatograms, in one of the cases, a small peak was more evident that could be interpreted as a missed mutation in the chromatogram evaluation. In the second, the chromatogram was not informative but, for both situations, we considered that we may be facing allelic fractions close to the limits of detection of the assay. In Uromonitor^®^ technical validation, the limit threshold was defined at more than 6.25% mutant sequences spiked in wildtype DNA [25,26]. Although the published technical data are conservative for assuring the positive detection in a commercial assay, from published data, TERTmonitor is already more effective in comparison to Sanger sequencing (20%) but still under the limits of next-generation sequencing (NGS) and digital PCR (dPCR) [36,37]. The greatest advantages of qPCR towards NGS and dPCR are the simplification of the method, the cost effectiveness, and, most importantly, the lack of expensive equipment; the latter is particularly relevant as, in a post-COVID-19 era, there are many qPCR machines and technicians who are very well trained. Another important consideration, not fully addressed in this study, is the difficulty to detect these *TERTp* mutations using Sanger sequencing. TERTmonitor uses a patented technology with mutant allele-specific primers and TaqMan^®^-based probes for real-time detection that allows for maximum sensitivity and specificity in the detection of these alterations. As a highly GC-rich region (~80%), it is extremely challenging to design primers and probes with high specificity and sensitivity in detecting mutations in the *TERTp*, particularly because this region forms complex and strong secondary structures that resist denaturation and interfere with primer annealing. Conventionally designed primers and TaqMan-MGB probes have failed, as they tend to form self- and cross-dimers as well as stem-loop (or hairpin) structures that impede the progress of the DNA polymerase along the template molecule. This leads to truncated PCR products and poor and/or unspecific amplification. In line with that, *TERTp* mutation detection by Sanger is often problematic, without clear results, requiring quite many replicates that increase the costs of the process [38,39]. 

From these study results, we pinpointed two cases, where an improved limit of detection was able to rescue apparently wildtype cases that had a small fraction of mutated alleles. The consideration that the Sanger sequencing was the gold standard and did not present a false result dragged the TERTmonitor to a lower performance; the opposite approach in the comparison would clearly increase TERTmonitor performance, but we chose not to make this consideration as we clearly established Sanger sequencing as the gold standard. Another important aspect is the low cellularity present in diffuse glioma samples that can benefit from increased sensitivity. Before treating patients whose imaging studies highly suggest glioma, a stereotactic biopsy is often performed but is frequently inaccurate in providing a correct diagnosis and is associated with additional risk and cost. Due to the tumour’s location, infiltrative growth, low cellularity, and frequently small stereotactic biopsy specimens, the histological confirmation of diffuse gliomas can be challenging [40]. Glioma-defining mutations allowed us, via a qPCR genotyping approach, to identify previously inconclusive intraoperative stereotactic biopsy samples, in the first available specimen [41]. The intraoperative diagnosis of diffuse glioma is critical to discriminate the tumour from non-neoplastic mimics and to set areas of neurosurgical resection. This is beneficial in terms of the point-of-care diagnosis in gliomas but also on other lesions defined by these highly recurrent mutations [41]. *TERTp* mutations have been identified in a large number of other cancers [8] and appear to be able to differentiate tumours from non-neoplastic tissue [41].

As in the original presentation of Uromonitor^®^, where multiple molecular targets are evaluated simultaneously, for gliomas, TERTmonitor can be expanded to include other biomarkers as IDH or targeting other events as the common hotspot mutations in pediatric settings, e.g., H3F3A and BRAF hotspots, as previously suggested by other authors [42]. Because of the inherent constraints associated with the CNS study tumour materials, along with the high prevalence of conservative techniques yielding limited and occasionally challenging histological samples, the inclusion of molecular data is notably propelled for the advance of tumour homogenous classification. In other models, we already have some experience, and we explored what *TERTp* mutations can offer as a biomarker that could be valuable in early detection [25,26] and using bodily fluids that, in this case, adapted to gliomas, could be the case of cerebrospinal fluid or liquid biopsies.

## 5. Conclusions

In conclusion, in this study, we evaluated the performance of a qPCR assay, TERTmonitor, in detecting −124 G>A and −146 G>A *TERTp* mutations in gliomas, while comparing it to Sanger sequencing, the reference method. The study accuracy was 97.8%, and the rare pitfalls are of no great difficulty to circumvent with a trained technician. The qPCR genotyping assay unlocks a higher sensitive analysis of *TERTp* mutations and suggests that this assay can be a potential tool for diagnosing and stratifying glioma patients, a condition where this biomarker has a pivotal role.

## Figures and Tables

**Table 1:** Glioma stratification by histotype and corresponding mutation frequencies.

	Sanger Sequencing, *n* (%)			TERTmonitor, *n* (%)		
	−124 G>A	−146 G>A	Total	−124 G>A	−146 G>A	Total
Pilocytic astrocytoma (*n* = 8)	0 (0.0)	0 (0.0)	0 (0.0)	0 (0.0)	0 (0.0)	0 (0.0)
Diffuse astrocytoma (*n* = 29)	4 (13.8)	0 (0.0)	4 (13.8)	4 (13.8)	1 (3.4)	5 (17.2)
Oligodendroglioma (*n* = 29)	11 (37.9)	5 (17.2)	16 (55.2)	11 (37.9)	5 (17.2)	16 (55.2)
Anaplastic oligodendroglioma (*n* = 37)	14 (37.8)	9 (24.3)	23 (62.2)	14 (37.8)	9 (24.3)	23 (62.2)
Glioblastoma (*n* = 76)	40 (52.6)	12 (15.8)	52 (68.4)	40 (52.6)	13 (17.1)	53 (69.7)
Gliosarcoma (*n* = 6)	3 (50.0)	3 (50.0)	6 (100.0)	3 (50.0)	3 (50.0)	6 (100.0)

## Data Availability

Patient data is unavailable due to ethical restrictions.
